# Right ventricular function and adaption after hemi-Fontan completion in children with hypoplastic left heart syndrome

**DOI:** 10.1186/1532-429X-18-S1-P161

**Published:** 2016-01-27

**Authors:** Bram Ruijsink, Hannah Bellsham-Revell, Kuberan Pushparajah, Reza Razavi

**Affiliations:** 1 grid.420545.2Paediatric Cardiology, Evelina's Childrens Hospital, Guy's and St Thomas NHS Foundation Trust, London, United Kingdom; 2 grid.13097.3c0000000123226764Division of Imaging Sciences & Biomedical Engineering British Heart Foundation Centre, King's College London, London, United Kingdom

## Background

The systemic right ventricle (RV) in patients with hypoplastic left heart syndrome (HLHS) is prone to failure. The complex geometry and contraction pattern of the systemic RV makes identification of patients at risk for failure challenging, especially in the light of radical changes in loading conditions during staged palliation. MRI allows for accurate quantification of cardiac volumes and provides assessment of the whole heart without restrictions in imaging planes. Recent advances in cardiac MRI analysis software allows for quantification of regional deformation from conventional balanced steady state free precession cine images.

We sought to investigate changes in ventricular function before and after the hemi-Fontan (HF).

## Methods

25 HLHS patients underwent cardiac MRI both before HF and before total cavopulmonary connection (TCPC). Global and regional strain parameters, mechanical dyssynchrony index (MDI) and cardiac volumes were obtained from all exams and compared pre- and post-HF. Circumferential, longitudinal and radial strain, strain rate, type of contraction pattern and MDI were correlated with volumetric indices and global systolic function in order to investigate possible mechanisms of RV dysfunction.

## Results

The volume unloading during HF surgery resulted in a decrease in end-diastolic and end systolic volumes (*p*<.05), with an increase in RV ejection fraction (EF) (*p*<.01). RV longitudinal strain did not significantly change from pre-HF to pre-TCPC whereas circumferential and radial strain increased resulting in a more circumferential rather than longitudinal dominant contraction pattern (longitudinal/circumferential strain ratio decreased; *p*<.01). Mechanical contraction became more synchronous; MDI pre-HF 11.4%, pre-TCPC 7.2% (*p*<.01). Both changes in global radial and circumferential strain correlated with EF (radial *r*=-.69, *p*<.01; circumferential *r=*0.74, *p*<.01) whereas longitudinal strain did not. The change in contraction pattern from longitudinal to circumferential dominant contraction was not correlated with a decline in ventricular EF (*r*=.16, *p*=.47). Mechanical dyssynchrony index decreased from pre-HF to pre-TCPC assessment but this was not correlated to an increase in EF.

## Conclusions

Changes in MRI derived indices of circumferential contraction (i.e. circumferential and radial strain) correlate with changes of global ventricular function in children with HLHS undergoing staged palliation. However, changes in longitudinal strain and mechanical dyssynchrony do not seem to correlate with EF. Serial assessment of circumferential contraction indices might be useful in the identification systemic right ventricular adaptation in response to staged surgical palliation in HLHS.

**Table 1:** Changes in MRI derived RV volumetric, global functional indices and contraction pattern between pre Hemi-Fontan and pre-total cavopulmonary connection exams.

	Pre-HF	Pre-TCPC	p-value
Indexed EDV (ml/m2)	95	81	0.02
Indexed ESV (ml/m2)	46	34	<.01
Ejection Fraction (%)	52	59	<.01
Cardiac Index (L/min/m2)	5.03	4.08	<.01
Longitudinal strain (%)	-15.2	-16.2	0.26
Radial strain (%)	26.3	37.1	<.01
Circumferential strain (%)	-15.1	-19.1	<.01
Long/circ strain ratio	1.04	0.84	<.01
Mechanical dyssynchrony index (%)	11.4	7.2	<.01

Pre-HF; pre hemi-Fontan, pre-TCPC; pre-total cavopulmonary connection, EDV; end diastolic volume, ESV; end systolic volume, SV; stroke volume. Mechanical dyssynchrony; standard deviation of time to peak radial systolic strain / duration of contraction.

**Figure 1:**
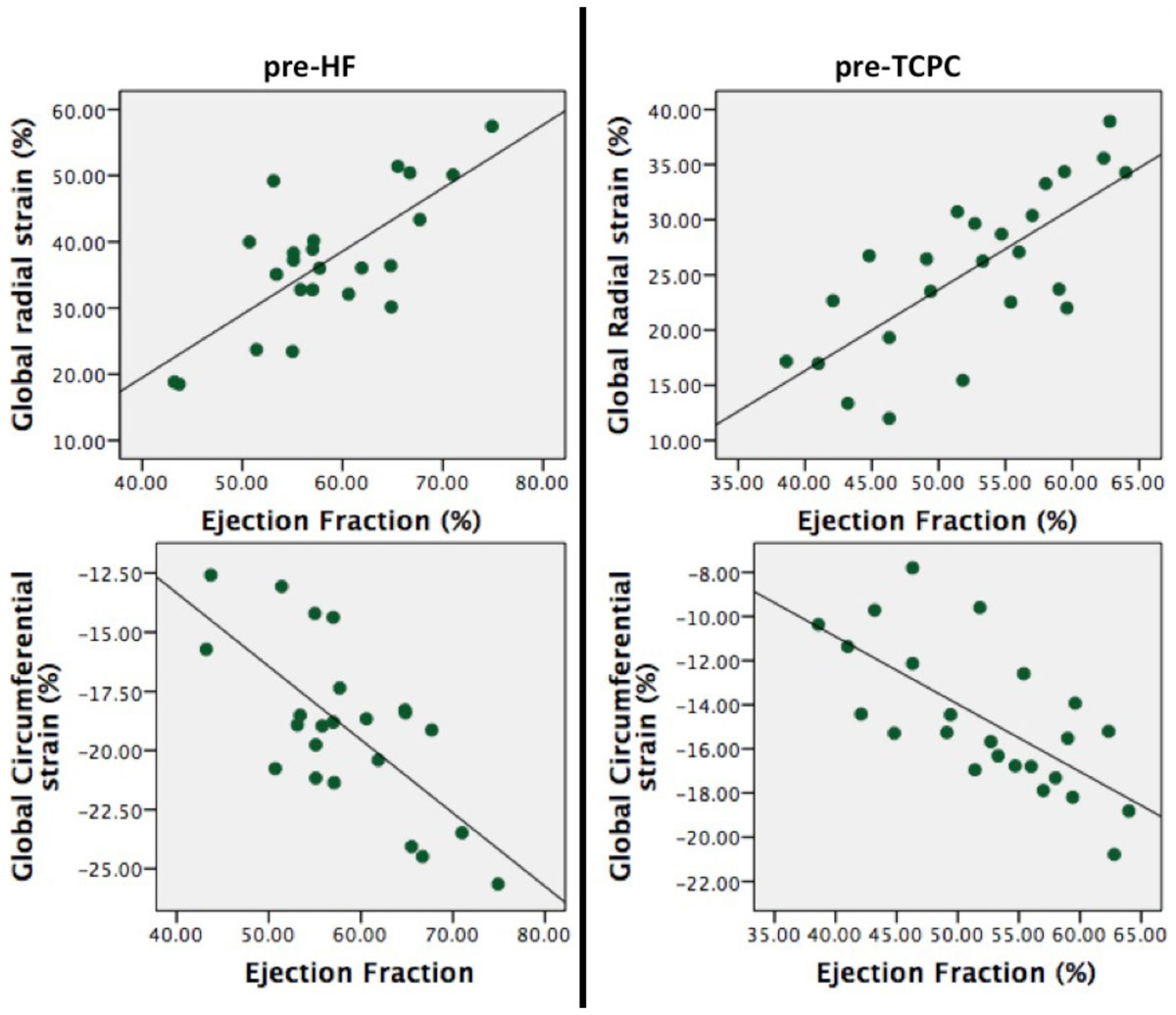
**Changes in global radial and circumferential strain in relation with ejection fraction.** HF; Hemi Fontan TCPC; total cavopulmonary connection

